# The local hypothalamic–pituitary–adrenal axis in cultured human dermal papilla cells

**DOI:** 10.1186/s12860-020-00287-w

**Published:** 2020-06-10

**Authors:** Eun Young Lee, You Jin Nam, Sangjin Kang, Eun Ju Choi, Inbo Han, Jinwan Kim, Dong Hyun Kim, Ji Hae An, Sunghou Lee, Min Ho Lee, Ji Hyung Chung

**Affiliations:** 1 Department of Biotechnology, CHA University, 5th Flr. CHA Bio Complex, 355, Pangyo-ro, Bundang-gu, Seongnam-si, Gyeonggi-do Korea; 2 Center for Non-Clinical Evaluation, CHA Advanced Research Institute, Seongnam, Korea; 3 Chabio F&C, Seongnam, Korea; 4 Department of Neurosurgery, CHA University, CHA Bundang Medical Center, Seongnam, Korea; 5 Department of Dermatology, CHA University, CHA Bundang Medical Center, Seongnam, Korea; 6 Department of Biomedical Technology, College of Engineering, Sangmyung University, Cheonan, Korea; 7 GF HERB, Nonsan, Chungnam Korea

**Keywords:** Corticotropin releasing factor, Hair loss, Human dermal papilla cells, Human hair follicle, Stress hormone

## Abstract

**Background:**

Stress is an important cause of skin disease, including hair loss. The hormonal response to stress is due to the HPA axis, which comprises hormones such as corticotropin releasing factor (CRF), adrenocorticotropic hormone (ACTH), and cortisol. Many reports have shown that CRF, a crucial stress hormone, inhibits hair growth and induces hair loss. However, the underlying mechanisms are still unclear. The aim of this study was to examine the effect of CRF on human dermal papilla cells (DPCs) as well as hair follicles and to investigate whether the HPA axis was established in cultured human DPCs.

**Results:**

CRF inhibited hair shaft elongation and induced early catagen transition in human hair follicles. Hair follicle cells, both human DPCs and human ORSCs, expressed CRF and its receptors and responded to CRF. CRF inhibited the proliferation of human DPCs through cell cycle arrest at G2/M phase and induced the accumulation of reactive oxygen species (ROS). Anagen-related cytokine levels were downregulated in CRF-treated human DPCs. Interestingly, increases in proopiomelanocortin (POMC), ACTH, and cortisol were induced by CRF in human DPCs, and antagonists for the CRF receptor blocked the effects of this hormone.

**Conclusion:**

The results of this study showed that stress can cause hair loss by acting through stress hormones. Additionally, these results suggested that a fully functional HPA axis exists in human DPCs and that CRF directly affects human DPCs as well as human hair follicles under stress conditions.

## Background

Hair follicles (HFs) consist of mesenchymal-derived dermal papilla cells (DPCs) and epithelial-derived root sheath cells, and the interaction between mesenchymal and epithelial tissues regulates hair development and the hair cycle [1, 2]. The hair cycle is divided into anagen, catagen, and telogen according to the morphology of the HFs. These cycles can be explained in terms of the growth phase, regression phase, and rest phase [3]. In addition, DPCs produce and secrete various cytokines that control hair development and the hair cycle [4]. Indeed, many researchers have used DPCs to study hair loss.

The hypothalamic–pituitary–adrenal axis (HPA axis) is a classical system response that plays a role in maintaining stress-related homeostasis. This essential response is activated by various factors, including physical, emotional, and neurological stresses [5]. Corticotropin releasing factor (CRF), adrenocorticotropic hormone (ACTH), and glucocorticoids are crucial stress-related hormones of the HPA axis [6]. Stress stimulates CRF production in the hypothalamus. After activation through the CRF receptor (CRFR), the anterior pituitary produces proopiomelanocortin (POMC)-derived ACTH, which produces cortisol (human) or glucocorticoids (rodent) as the main end effectors of the HPA axis in the adrenal cortex [7–10]. Cortisol acts as a negative feedback system to suppress the release of CRF. Thus, the hormone levels in the blood are maintained at a constant level [11].

CRF, composed of 41 amino acids, is the main trigger of the HPA axis and also plays an important role in the stress response of vertebrates. Activation of adenylyl cyclase and upregulation of cyclic AMP levels occur when CRF interacts with CRFR1 or CRFR2, membrane receptors of the class B subtype of G protein coupled receptors (GPCRs), in response to these receptors [12, 13]. As a result, protein kinase A (PKA) and cAMP response element binding protein (CREB) phosphorylate downstream targets in the cytoplasm and nucleus, respectively [14].

The human skin is the largest organ and has abundant HFs, except on the palms, soles, and nails [1]. There are many reports of cutaneous HPA axis homologues in the skin [6]. Skin expresses all elements of the HPA axis, including CRF, POMC, ACTH, and β-endorphin, with the corresponding receptors [9, 15]. These results indicate that these molecules play a role in the skin. A previous study showed that human HFs have their own peripheral HPA axis. Additionally, human HFs secrete stress-related hormones, including CRF, ACTH, and glucocorticoids. The human hair bulb is the target of CRF as well [16]. Moreover, CRF inhibited hair growth and induced apoptosis of hair matrix keratinocytes in cultured human HFs [17, 18]. Interestingly, hair regrowth was observed in a CRF-overexpressing mouse model after treatment with a CRF receptor antagonist [19].

Recently, studies have specifically examined the interactions among stress hormones, the neuroendocrine system and skin diseases, such as hair loss [20–24]. Stress changes the hair cycle and induces hair loss. However, the underlying biochemical mechanisms of stress-induced hair loss are still unclear.

The aims of this study were to determine the CRF-induced modulations in human DPCs that have a major role HFs and investigate whether the local HPA axis is functional in DPCs. Through these analyses, we will elucidate the mechanism of stress-induced hair loss.

## Results

### CRF, a stress hormone, inhibited hair shaft elongation and induced early catagen transition in human HF organ culture

**Fig. 1:**
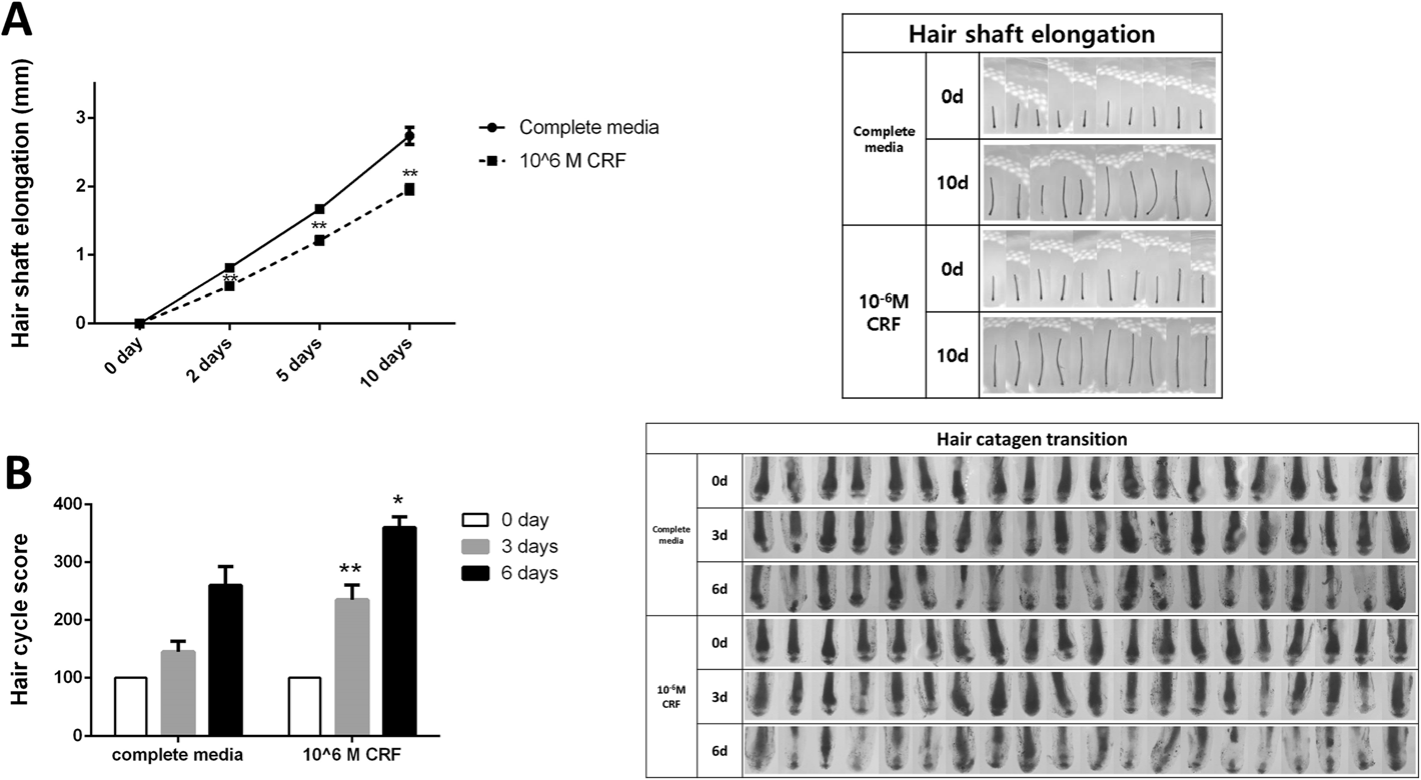
Effect of CRF on human HF organ culture. Human HFs were incubated with or without CRF at 10^− 6^ M for 10 days. **a** Hair shaft elongation was measured on days 0, 2, 5, and 10 using a stereomicroscope. The images were taken at 10× magnification. **b** Hair cycles of cultured human HFs were scored on day 0, 3 and 6 according to the hair cycle scoring guidelines. The images were taken at 50× magnification. *p* values of HF elongations compared with those of the control group on the same day, *p*-values were determined by t-tests, *: *p* < 0.05, **: *p* < 0.01

To confirm the deleterious effects of CRF on HFs, we exposed anagen-phase human HFs to CRF, and hair shaft elongation and hair cycle transition were monitored. CRF significantly suppressed hair shaft elongation. The hair shaft was decreased by approximately 30% in the CRF treatment group compared with that in the negative control group at 2, 5, and 10 days (Fig. 1a).

The hair cycle score is an arbitrary grading method based on the morphological characteristics of each HF. Anagen VI HFs were ascribed an arbitrary value of 100, early catagen HFs were given a score of 200, mid-catagen HFs were given a score of 300, and late catagen HFs were given a score of 400; these values were used to calculate the hair cycle score [25–27]. The hair cycle score was significantly higher in the CRF-treated group (235 ± 25.4 and 360 ± 18.4) than in the control group (145 ± 18.5 and 260 ± 32.8) at 3 and 6 days, respectively, indicating that CRF induced premature catagen transition (Fig. 1b).

### Human DPCs express CRF receptors and respond to CRF

**Fig. 2:**
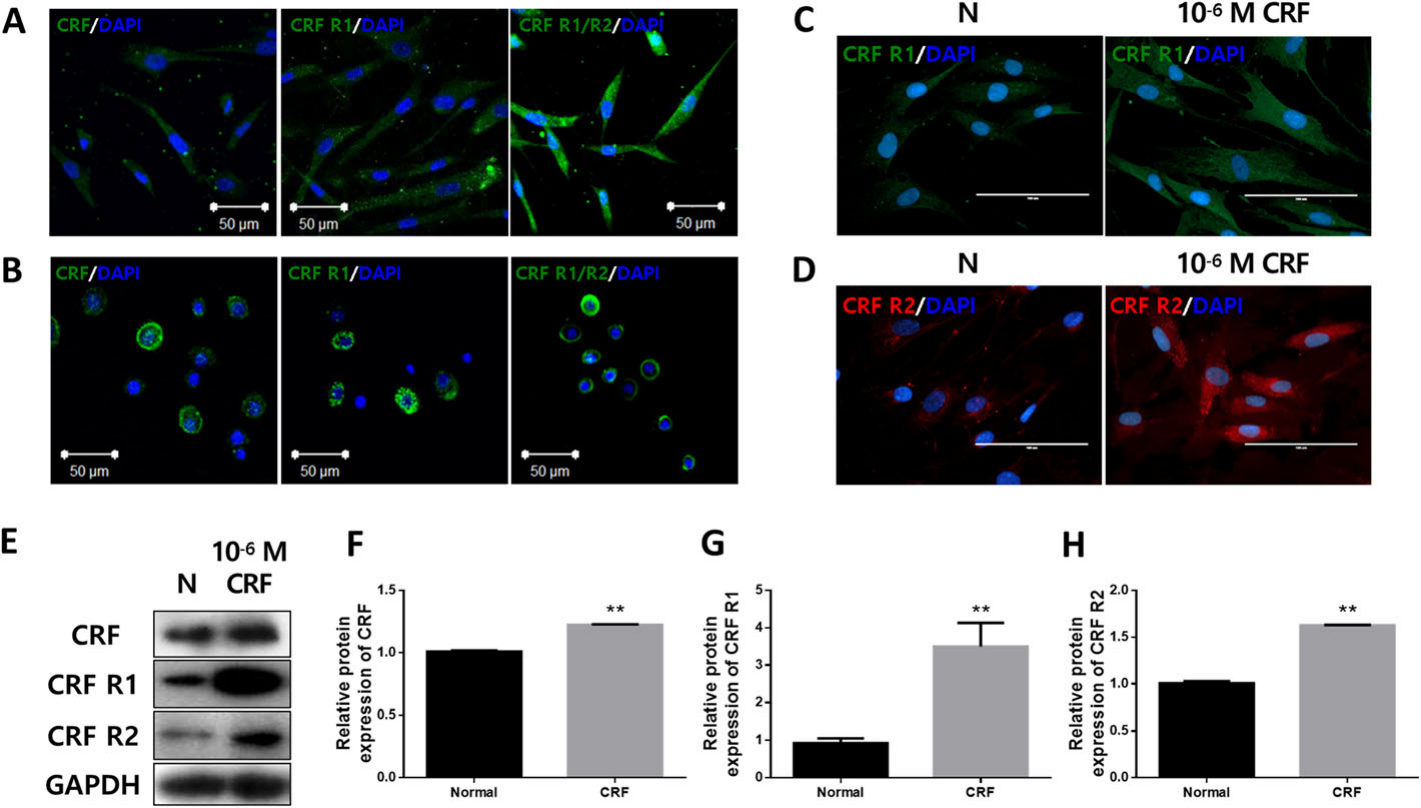
Response of cultured human DPCs to CRF. The endogenous expression of CRF and its receptors in cultured human DPCs (**a**) and ORSCs (**b**) (scale bars = 50 μm, blue: DAPI, green: IgG, CRFR1, or CRFR1/2). **c**, **d** Human DPCs were exposed to 10^− 6^ M CRF for 24 h and assessed fluorescence intensity of CRFR1 and CRFR2 using immunocytochemistry (scale bars = 100 μm, blue: DAPI, green: IgG, CRFR1, red: IgG, CRFR2) and **e** confirmed using western blots. **f**, **g**, **h** The bands were quantified using ImageJ. *p* values compared with the normal group by t-tests, *: *p* < 0.05, **: *p* < 0.01

To determine whether CRF can affect the number of cells comprising the HFs, we analyzed the expression of CRF receptors in cultured human DPCs and outer root sheath cells (ORSCs). Endogenous expression of CRF and CRF receptors was detected by the immunocytochemistry method in both human DPCs and ORSCs (Fig. 2a, b). Interestingly, the CRFR1 fluorescence intensity was increased with 10^− 6^ M CRF treatment (Fig. 2c). Also, the CRFR2 fluorescence intensity was increased in human DPCs after exposure to 10^− 6^ M CRF for 24 h (Fig. 2d). Increased expression of CRF and its receptors was confirmed by western blot analysis. Both CRFR1 and CRFR2, as well as CRF, were endogenously expressed and increased by CRF treatment in human DPCs (Fig. 2e, f, g, h).

### CRF inhibited proliferation through cell cycle arrest in cultured human DPCs

**Fig. 3:**
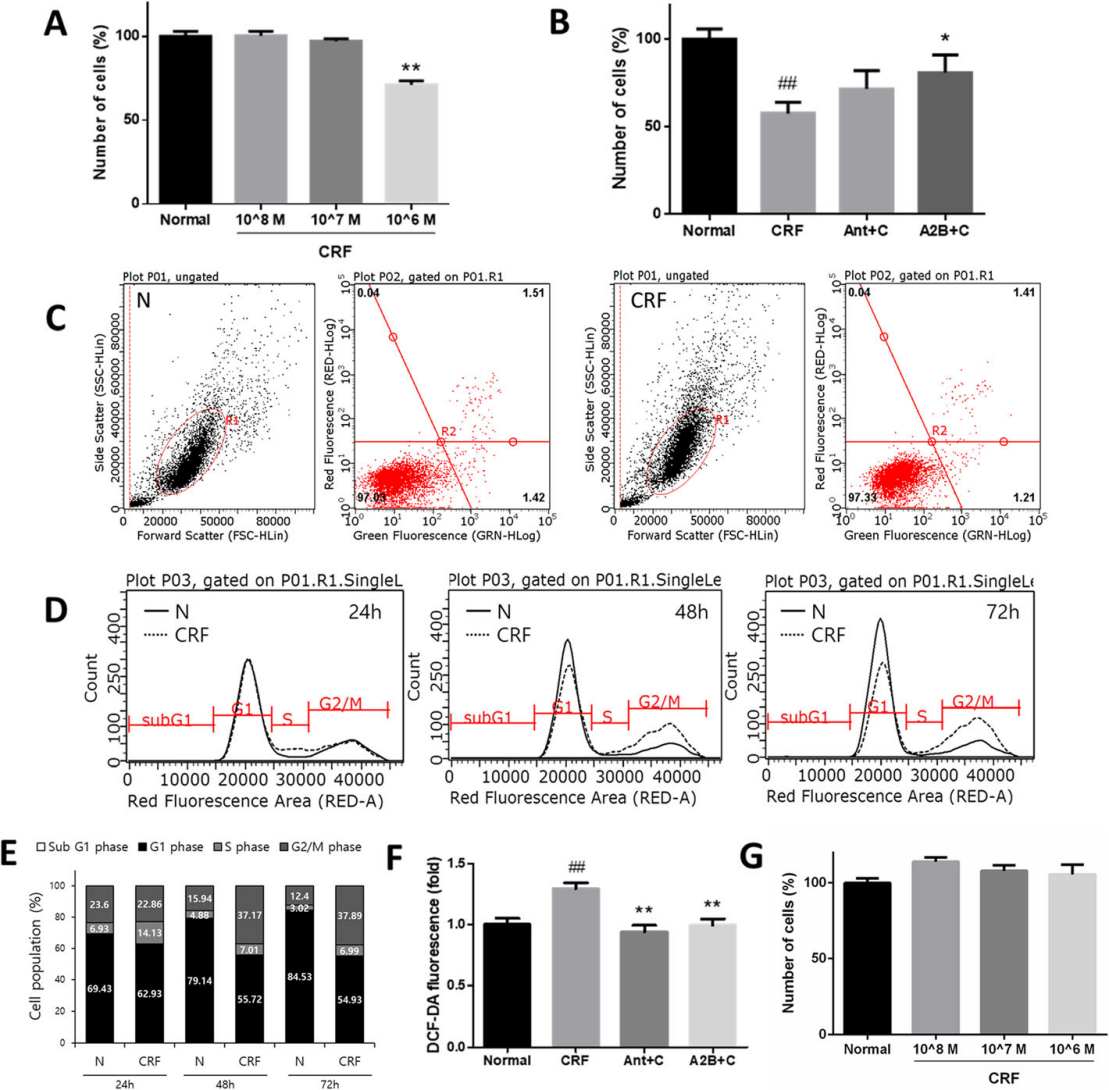
The effects of CRF on the proliferation, apoptosis, and cell cycle of human DPCs and human ORSCs. **a** Dose-dependent effect of CRF on the proliferation of human DPCs. Cells were exposed to CRF at concentrations of 10^− 8^ M, 10^− 7^ M, and 10^− 6^ M for 48 h. **b** The protective effect of CRF antagonists on the proliferation of human DPCs. The cell number was determined using an EZ-Cytox kit. **c** The effect of CRF on apoptosis in DPCs. Samples were stained with Annexin V FITC and PI. **d** Cell cycle analysis of human DPCs at 24, 48, and 72 h after CRF treatment using flow cytometry and **e** distribution of the cell cycle. **f** ROS generation after CRF treatment measured using H_2_DCFDA. CRF antagonists normalized the production of ROS on cultured human DPCs. **g** Dose-dependent effect of CRF on the proliferation of human ORSCs. Cells were exposed to CRF at concentrations of 10^− 8^ M, 10^− 7^ M, and 10^− 6^ M for 48 h. Ant; antalarmin, A2B; astressin 2-B, C; CRF. *p* values compared with the normal group by t-test, ##: *p* < 0.01. *p* values compared with the CRF treatment group by t-tests, *: *p* < 0.05, **: *p* < 0.01

For selection of the experimental CRF concentration, we exposed human DPCs to various concentrations of CRF, and the cells were quantified after 2 days. A significant difference was observed in total cells between the CRF group (at a concentration of 10^− 6^ M) and the control group, and this change was reversed by antagonists of the CRF receptor (Fig. 3a, b).

To determine whether the changes in the CRF treatment group were caused by cell death or by inhibition of cell proliferation, we exposed cells to 10^− 6^ M CRF for 48 h and stained them with Annexin V FITC and PI. In the untreated control sample, the majority (97.03%) of the cells were viable and nonapoptotic, and the CRF-treated cells showed no change in viability (97.33%) (Fig. 3c). This result suggests that CRF did not influence cell survival or apoptosis but inhibited cell proliferation.

To further determine how CRF suppressed the proliferation of human DPCs, we performed FACs analysis of the cell cycle. Compared with the negative control treatment, treatment with 10^− 6^ M CRF resulted in a 7.2% increase in the S phase cells at 24 h. Ultimately, CRF resulted in 21.2 and 25.5% increases in the G2/M phase cells at 48 h and 72 h, respectively. Interestingly, we did not observe any apoptotic cells (sub-G1 fraction) up to 72 h after the CRF treatment (Fig. 3d, e).

Because accumulation of ROS was implicated in cell cycle arrest [28], the ROS levels were monitored in CRF-treated cells by DCFDA fluorescence analysis. CRF increased the fluorescence signal by approximately 30% compared to that of the control group (Fig. 3f). Additionally, antalarmin and astressin 2-B normalized the DCF fluorescence levels. These data indicated that CRF induced ROS in cultured human DPCs.

In order to verify if CRF affects the proliferative activities of cultured ORS cells, cell viability assay was performed using EZ-cytox kit. Cells were treated with various concentrations of CRF for 2 days. As a result, CRF have no direct effects on proliferation of cultured ORS cells (Fig. 3g).

### The expression levels of anagen-related cytokines were downregulated by CRF in cultured human DPCs

**Fig. 4:**
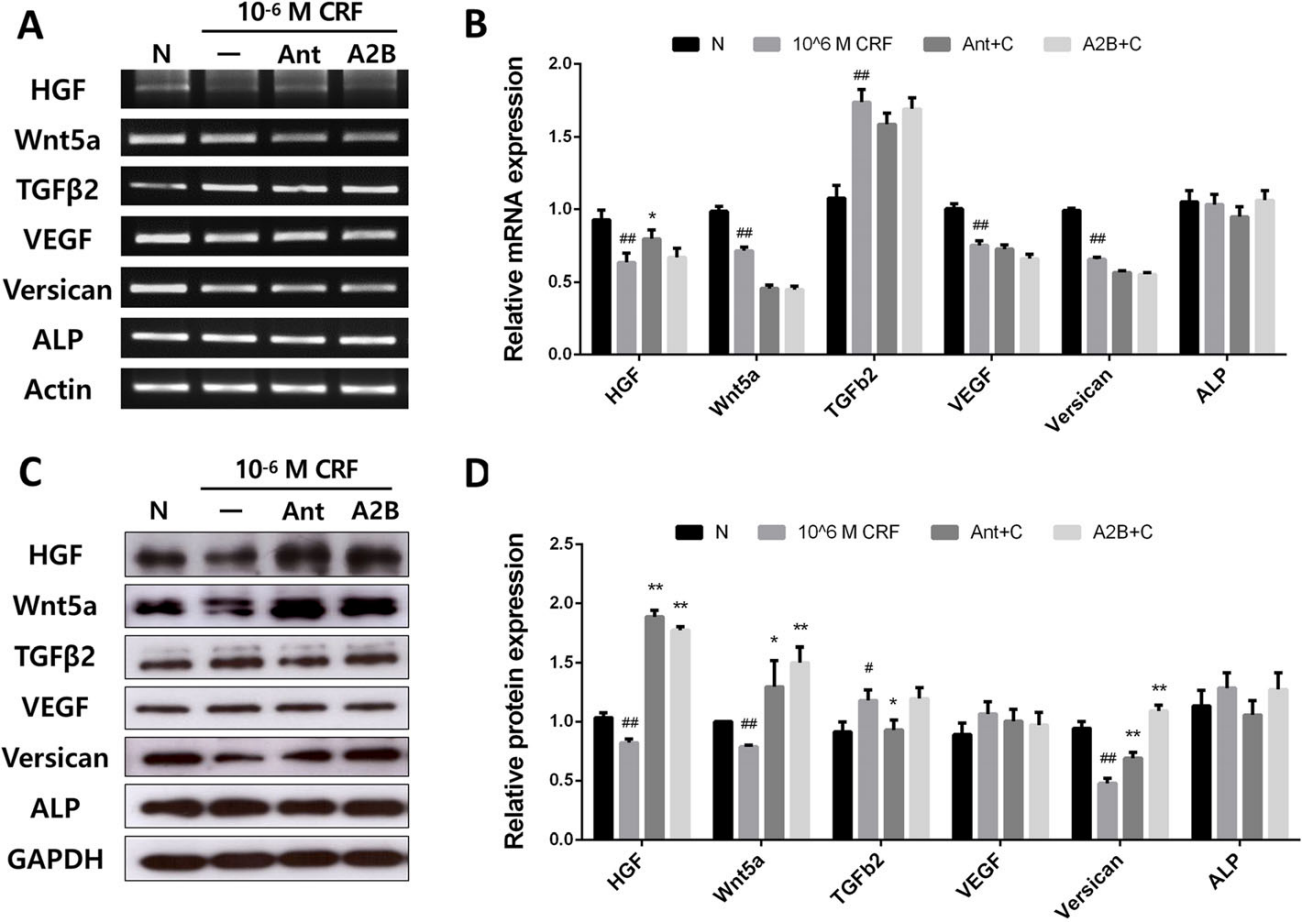
The expression levels of anagen-related cytokines in cultured human DPCs with CRF treatment. Antagonists normalize the expression levels of anagen-related cytokines in cultured human DPCs with 10^− 6^ M CRF treatment. **a**, **b** mRNA expression levels (RT-PCR). The bands were quantified using ImageJ. **c**, **d** Protein expression levels (western blot). The bands were quantified using ImageJ. Ant; antalarmin, A2B; astressin 2-B, C; CRF. *p* values compared with the control group by t-tests, #: *p* < 0.05, ##: *p* < 0.01. *p* values compared with the CRF treatment group by t-tests, *: *p* < 0.05, **: *p* < 0.01

RT-PCR and western blot analyses of cultured human DPCs were performed to determine whether CRF affects cytokines, which are typically produced and released by DPCs during the anagen phase. CRF significantly changed the mRNA levels of HGF, Wnt5a, TGFβ2, VEGF, and versican, but the CRFR1 antagonist antalarmin significantly affected only HGF (Fig. 4a, b). The protein levels of these cytokines, including HGF, Wnt5a, TGFβ2, and versican, were also modulated by CRF treatment and were effectively restored with antalarmin or astressin 2-B pretreatment (Fig. 4c, d).

### The local HPA axis in cultured human DPCs

**Fig. 5:**
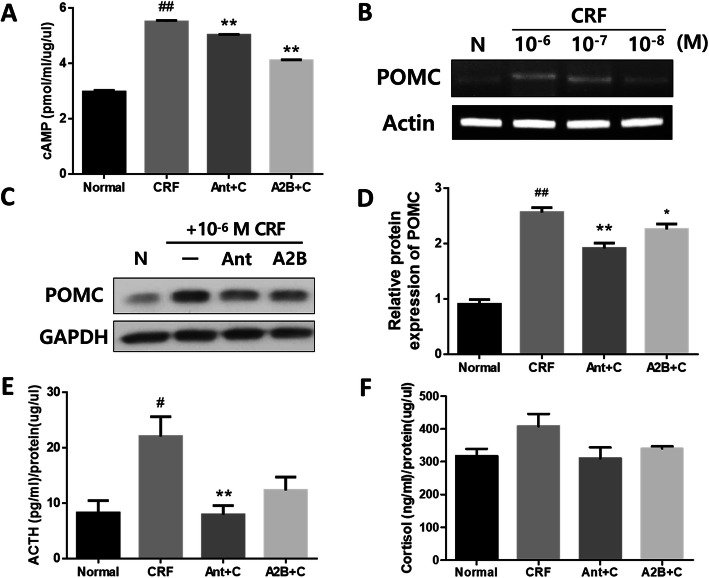
The local HPA axis in cultured human DPCs. **a** Human DPCs were exposed to 10^− 6^ M CRF with antagonists for 1 h, and the cAMP level was measured using an ELISA kit. *p* values compared with the control group by t-tests, #: *p* < 0.05, ##: *p* < 0.01. *p* values compared with the CRF treatment group by t-tests, *: *p* < 0.05, **: *p* < 0.01. **b** POMC mRNA levels with CRF treatment at 6 h (RT-PCR). **c** POMC protein levels with CRF and antagonist treatment at 6 h (western blot). **d** The bands were quantified using the ImageJ program. **e** ACTH concentrations at 24 h. **f** Cortisol levels at 48 h. Ant; antalarmin, A2B; astressin 2-B, C; CRF. *p* values compared with the control group by t-tests, #: *p* < 0.05, ##: *p* < 0.01. *p* values compared with the CRF treatment group by t-tests, *: *p* < 0.05, **: *p* < 0.01

To confirm the downstream events following CRF-receptor binding, we monitored the levels of a second messenger, cAMP, after CRF treatment. Following CRF treatment, the cAMP level was approximately two times (5.51 ± 0.05 pmol/ml/μg/μl) that of the control (2.97 ± 0.06 pmol/ml/μg/μl). Additionally, the cAMP levels were significantly decreased by antalarmin (5.03 ± 0.03 pmol/ml/ug/ul) and astressin 2-B (4.11 ± 0.03 pmol/ml/μg/μl). Therefore, we confirmed that the CRF effects occurred inside human DPCs (Fig. 5a).

To determine whether the HPA axis was fully functional in cultured human DPCs, we administered antagonists to cells incubated with 10^− 6^ M CRF and analyzed the expression levels of POMC, ACTH, and cortisol. The mRNA and protein levels of POMC were upregulated by CRF treatment (Fig. 5b, c). In particular, the protein level increased almost 2.5-fold. The increased POMC protein was significantly downregulated by both antalarmin and astressin 2-B treatment (Fig. 5c, d). In addition to these changes, the secretion of ACTH was stimulated by CRF (2.7-fold) in cultured human DPCs. Antalarmin significantly recovered the ACTH concentration, but astressin 2-B showed a tendency to decrease ACTH, and the effect was not significant (Fig. 5e). Furthermore, CRF slightly increased the cortisol level (1.28-fold) in cultured human DPCs (Fig. 5f). The increased cortisol level, though not significant, was reduced by antalarmin and astressin 2-B treatment. Overall, these findings indicate that cultured human DPCs have a complete local HPA axis.

## Discussion

Recently, hair loss has increased in women and young people. This hair loss is believed to be caused by various stressors rather than by genetic factors. Thus, the accumulation of data on the effects of stress on HFs and their constituent cells is important for treatment of hair loss [8, 29, 30]. However, knowledge of the relationship between stress hormones and hair loss is limited. In particular, CRF is an important hormone of the HPA axis in the peripheral stress response. While several reports have identified the role of stress hormones such as CRF, ACTH, and cortisol in hair loss in in vivo models or human HF organ culture, the exact cellular mechanisms have not been studied [16, 19, 31].

In this study, CRF stimulation significantly inhibited hair shaft elongation and induced the early catagen transition of human HFs in culture. These results are consistent with previous reports about stress-induced hair loss [8, 17]. However, these studies focused on apoptosis or proliferation of HFs in ex vivo. To further elucidate the mechanisms of stress-induced hair loss, we have to investigate how CRF acts on cells that constitute the HFs, especially DPCs, which are specialized mesenchymal cells in HFs and are believed to play important roles in hair growth and the hair cycle. As a result, the expression of CRF receptors was identified in cultured human DPCs, which is the first in vitro report of this finding according to our knowledge. In addition, the expression of CRF receptors was elevated when CRF was added to cultured human DPCs. These findings suggested that the CRF receptors were highly sensitive in cultured human DPCs.

To determine how CRF affects cultured human DPCs, we performed a cell proliferation assay. The results showed that CRF significantly changed the morphology (data not shown) and inhibited the increase in cell numbers of the cultured human DPCs. These data indicated that CRF inhibited proliferation or induced apoptosis. Therefore, to clarify the cause of the decreased cell viability, we stained the cultured human DPCs with Annexin V FITC and PI. However, there were no significant changes in apoptosis. Interestingly, the DNA contents of the cultured human DPCs were changed by CRF. The G2/M phase ratio was increased by CRF stimulation for up to 72 h. In addition, ROS production was accelerated by CRF. Some reports have shown that ROS accumulation induces DNA damage, resulting in cell cycle arrest. Cellular senescence is a permanent cell cycle arrest induced by stress, including oxidative stress and DNA damage [32, 33]. Other studies showed that premature cellular senescence caused by oxidative stress in DP compromised the interaction between the epithelium and DP cells, and the above phenomenon was observed mainly in androgenetic alopecia patients [34, 35]. Although a further study on cell aging should be carried out, above data suggest that CRF leads to the production and accumulation of ROS in cells, thereby arresting cells in G2/M phase, which may ultimately could lead to cell senescence.

In cultured human DPCs, CRF downregulated various cytokines, including HGF, Wnt5a, VEGF, and versican, which are known to be expressed in the anagen phase and regulate hair growth. Both the active form of HGF and VEGF are essential paracrine factors that promote hair growth [36, 37]. Additionally, Wnts play an essential role in maintaining both hair inductive activity and anagen phase [38]. Versican is related to the development of pathologic processes such as HF cycling and cancer [39]. In addition, the mRNA and protein levels of TGFβ2 were increased with CRF treatment. TGFβ2 is an essential component of catagen induction in the human hair cycle [40].

Overall, CRF, a stress hormone, seems to induce a senescent state in cultured human DPCs through the following process: CRF causes G2/M arrest by ROS production and accumulation, which can lead to cell aging. In addition, CRF reduces the expression of anagen-related cytokines and promotes catagen-related cytokines, allowing HFs to enter the regression phase.

When CRF is coupled to its receptors, adenylate cyclase and phospholipase C are activated, and then cAMP is induced [41, 42]. To confirm the initiation of CRF signals in cultured human DPCs, we detected cAMP accumulation after CRF treatment. These data confirmed that CRF affects human DPCs through its receptors.

**Fig. 6:**
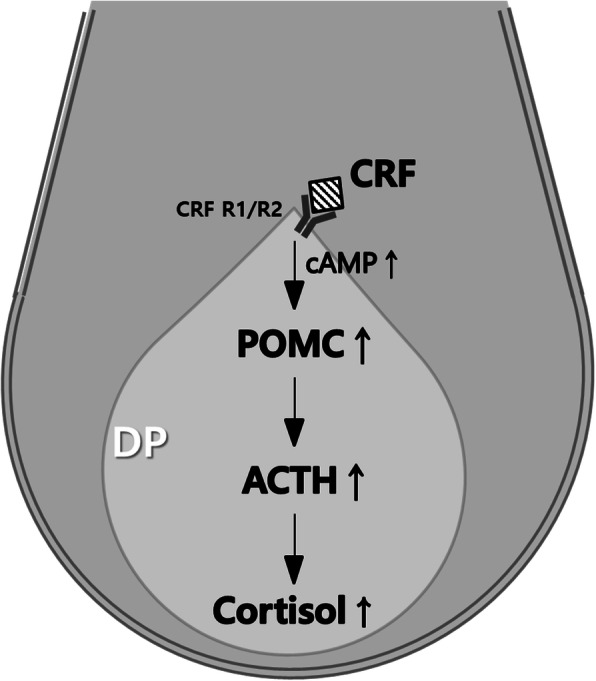
The possible mechanism of early catagen transition induced by the stress hormone CRF. DP; dermal papilla

Previous studies have detected a local HPA axis in human HF [17, 18, 43]. Fascinatingly, all data from in vitro system suggest that local HPA axis exists in cultured human DPCs. POMC, a precursor peptide of ACTH, was increased by CRF treatment at both the mRNA and protein levels. The levels of ACTH and cortisol were also increased when cultured DPCs were stimulated by CRF. A complete and functional local HPA axis might be present in cultured human DPCs (Fig. 6).

CRF receptors were confirmed to be expressed in the cultured ORS cells, and thus, the effects of CRF were investigated in these cells. CRF appeared to had no effect on the proliferation of ORS cells was not affected by CRF treatment, while apoptotic responses caused by CRF have been reported in several in vivo studies [17]. Nevertheless, since CRF inhibits hair shaft growth and induces premature catagen transition in hair organ culture it seems to be necessary to study about the direct and indirect effects of CRF on ORS cells. Further research, such as coculture studies of ORS cells and DPCs, will be required for an improved understanding of the complex dynamics of HFs. Investigation of the mesenchymal-epithelial interaction within the HFs will address this issue.

In this study, CRF inhibits hair growth and induces early catagen transition in human HF organ culture. In cultured DPCs derived from human HF, CRF causes G2/M arrest by ROS production and accumulation. CRF reduces the expression of anagen-related cytokines and promotes catagen-related cytokines. CRF seems to lead to the senescent state in cultured human DPCs. Furthermore, CRF induces the secretion of POMC, ACTH, and cortisol in vitro, indicating that local HPA axis also exists not only in HFs but also in cultured human DPCs. Although in vitro assay was performed to explain the hair loss caused by CRF, paradoxically, without in vivo data, the interpretation of these results is limited and careful because all the data came from in vitro culture cells. Nevertheless, this work may add to the possibility that CRF can be induce hair loss through induction of cellular senescence and modulation of hair cycle-related cytokines in the stress-induced hair loss mechanism.

## Conclusions

All data from cultured human DPCs derived from HFs suggest that a fully functional local HPA axis is present in cultured DPCs. Therefore, it seems that CRF can directly affects human DPCs as well as human HFs under stress condition. This study helped improve our understanding of the mechanisms of stress hormones leading to hair growth inhibition and hair loss.

## Methods

### Human HF organ culture and hair cycle scoring

Human scalp skin specimens were obtained from dermatology patients after obtaining informed consent, following the Declaration of Helsinki principles. The study was approved by the Institutional Review Board of the CHA Bundang Medical Center (IRB No. CHAMC 2018–09-009).

Anagen human HFs were isolated by microdissection and maintained in William’s E medium (WelGENE, South Korea) supplemented with 10 μg/ml insulin (Sigma-Aldrich, St. Louis, USA), 10 ng/ml hydrocortisone (Sigma-Aldrich), 20 mM HEPES (Invitrogen-Gibco-BRL, Grand Island, NY), and 1× antibiotic-antimycotic (Invitrogen-Gibco-BRL) for 1 day.

Isolated human HFs were exposed to 10^− 6^ M CRF. On every second or third days, the medium was replaced, and human HFs were photodocumented. The hair cycle stages of cultured human HFs were scored every day according to the hair cycle scoring guidelines [25–27].

### Cell culture and treatment

Three individual patients originated cells were used for the studies. Human DPC culture was established according to the method of Warren [44] with modifications. Cells were cultured in CnT Basal medium 2 containing two supplements (CnT-05.A and CnT-05.B) (CELLnTEC, Huissen, The Netherlands) with 1× antibiotic-antimycotic and incubated at 37 °C under 5% CO_2_.

For human ORSC culture, HFs were isolated from the dermis and attached to dishes coated with collagen type I. Outgrowing ORSCs were cultured in Keratinocyte SFM (Invitrogen-Gibco-BRL) at 37 °C under 5% CO_2_.

Cells were exposed to varying concentrations of CRF for up to 72 h at 37 °C. Materials including receptor antagonists were added 1 h before hormone treatments. Antalarmin (Sigma-Aldrich) and astressin 2-B (Sigma-Aldrich) were used as CRFR1- and CRFR2-specific antagonists, respectively.

### Immunocytochemistry

Cells were fixed with 4% formaldehyde for 10 min, washed with PBS and blocked with 5% BSA in PBS for 1 h. The slides were incubated with each primary antibody in blocking solution. After the cells were washed, they were labeled with secondary antibody with fluorescent dye for 1 h. Then, the cells were immediately mounted with DAPI solution and visualized with fluorescence microscopy (Thermo Fisher Scientific, USA). The antibodies used in immunocytochemistry are listed in Table 1.

**Table 1:** Antibodies used for the immunocytochemistry and western blot

Protein	Antibody company	Catalog number
GAPDH	Abfrontier	LF-PA0018
Wnt5a	Abcam	ab72583
VEGF	Abcam	ab46154
HGF	Abcam	ab83760
TGFβ2	Abcam	ab36495
Versican	Abcam	ab19345
ALP	Abcam	ab108337
POMC	Abcam	ab32893
CRF R1	Abnova	PAB7539
CRF R2	Abcam	ab167379
CRF R1/R2	Santa Cruz Biotechnology	sc-1757
CRF	Abcam	Ab8901

### Cell proliferation

The changes in cell number were assessed using an EZ-Cytox assay kit (Daeil Lab Service, Korea) according to the manufacturer’s instructions. Human DPCs were seeded at a density of 7000 cells per well in 48-well plates. After attachment, the cells were treated with each test material for 3 days. Medium was replaced with supplement-free CnT basal medium with 20 μl/well of EZ-Cytox solution. After 2 h, the absorbance was measured at 450 nm using a Synergy H1 Multi-Mode Reader (Biotek, USA). The optical density (OD) of each well was used to calculate the cell number based on the authentic standard curves.

### Measurement of intracellular reactive oxygen species (ROS)

Cells were washed with DPBS (WelGENE) and stained with 2′,7′-dichlorodihydrofluorescein diacetate acetyl ester (H_2_DCFDA, Thermo Fisher Scientific) for 30 min at 37 °C in dark conditions. Guava EasyCyte (Merck Millipore, Germany) was used for analysis.

### Cell cycle analysis

Cells were washed with DPBS (WelGENE), fixed with 80% cold ethanol for 2 h, centrifuged to remove the ethanol, and washed with cold DPBS 2 times. Cells were stained with PI/RNase in DPBS containing 1% FBS (HyClone, USA) for 30 min at 37 °C. Guava EasyCyte (Merck Millipore) was used to analyze the cell cycle.

### Apoptotic cell analysis

CRF-exposed cells were analyzed using the FITC Annexin V Apoptosis Detection Kit (BD Biosciences, San Jose, CA) according to the manufacturer’s instructions. Annexin V is used to identify apoptotic cells, and PI is used to distinguish viable from nonviable cells. Guava EasyCyte (Merck Millipore) was used for analysis.

### Semiquantitative RT-PCR

Total RNA was extracted from human DPCs using an RNA/protein one step kit (MACHEREY-NAGEL, Dueren, Germany) according to the manufacturer’s protocol. cDNA was synthesized from 2 μg of total RNA using reverse transcriptase, oligo dT and dNTPs and was used as the template for PCR. The cDNA was amplified in a PCR reaction containing 10 pM of specific primers using a Veriti 96 Well Thermal Cycler (Applied Biosystems, USA). Amplification consisted of 28 ~ 35 cycles as follows: denaturation at 95 °C for 5 min, annealing at 56.2 ~ 62 °C for 30 s and a final extension at 72 °C for 5 min. The PCR products were separated by 2% agarose gel electrophoresis and visualized with a Gel Doc™ EX Imager (Bio-Rad, USA). All primers used are described in Table 2.

**Table 2:** Primers used for the RT-PCR

Gene	PCR primer sequence	Product size (bp)
Actin	F 5′- GCG TGA CAT TAA GGA GAA GC -3′	174
	R 5′- AGG AAG GAA GGC TGG AAG A -3′	
Wnt5a	F 5′- CCA CAC AAG ACC TGG TCT ACA TC -3′	172
	R 5′- GTC TGC ACG GTC TTG AAC TG -3′	
VEGF	F 5′- TAC CTC CAC CAT GCC AAG T -3′	343
	R 5′- TGC ATT CAC ATT TGT TGT GC -3′	
HGF	F 5′- TGC AGA GGG ACA AAG GAA AAG -3′	378
	R 5′- GAA CTC CAG GGC TGA CAT TTG -3′	
TGFβ2	F 5′- AGG GTA CAA TGC CAA CTT CTG -3′	144
	R 5′- GGT TCT AAA TCT TGG GAC ACG -3′	
Versican	F 5′- TGA CTG TGG ATG GGG TTG TG -3′	141
	R 5′- GCG TCA CAC TGC TCA AAT CC -3′	
ALP	F 5′- CCA AGG ACG CTG GGA AAT CT -3′	172
	R 5′- TAT GCA TGA GCT GGT AGG CG -3′	
POMC	F 5′- CCC CTA CAG GAT GGA GCA CT -3′	116
	R 5′- GGC GTT TTT GAA CAG CGT CA -3′	

### Western blot analysis

Total protein was extracted from human DPCs using an RNA/protein one step kit (MACHEREY-NAGEL) according to the manufacturer’s protocol. The proteins were separated by electrophoresis in 10% SDS-PAGE gels and transferred to 0.2 μm pore size PVDF membranes (Millipore, Bedford, MA, USA). The membranes were blocked with 5% skim milk in Tris buffered saline-Tween 20 (TBST 0.1% Tween 20) for an hour and then incubated with each primary antibody overnight at 4 °C. After three washes, the membranes were incubated with secondary antibody for 1 h. After the samples were washed 5 times, immunoreactive protein was visualized with Clarity™ Western ECL Substrate (Bio-Rad) and detected using Amersham Hyperfilm™ ECL (GE Healthcare, UK). Experiments were performed three times. The antibodies used in the western blot analyses are listed in Table 1.

### cAMP assay

Human DPCs were incubated (5 × 10^5^ cells/100 mm dish) for 30 min at 37 °C in media containing 0.5 mM of phosphodiesterase inhibitor and 3-isobutyl-1-methylxanthine (IBMX) (Sigma-Aldrich). Then, CRF was added to media containing 0.5 mM of IBMX, and the cells were incubated for 1 h at 37 °C. The cAMP concentration was measured in cultured human DPCs using a Cyclic AMP ELISA Kit (Cayman, Michigan, USA) according to the manufacturer’s protocol. A Synergy H1 Multi-Mode Reader (Biotek) was used to measure the cAMP concentrations, and the concentration was calculated from the standard curve.

### ACTH assay

Cells were seeded at a concentration of 3 × 10^5^ cells per 100 mm dish. After attachment, the cells were treated with CRF receptor antagonists and then exposed to CRF. ACTH was measured with an ACTH ELISA kit (MD Bioproducts, Switzerland) according to the manufacturer’s protocol. The absorbance was measured with a Synergy H1 Multi-Mode Reader (Biotek). The OD of each well was used to calculate the ACTH concentration based on the authentic standard curves.

### Cortisol assay

Cells were seeded at a concentration of 3 × 10^5^ cells per 100 mm dish. Cells were pretreated with antalarmin and astressin 2-B for 1 h. CRF was added to the media. Cells were collected and washed with cold PBS and then lysed with lysis buffer. Cortisol levels were measured in cultured human DPCs using a Parameter cortisol kit (R&D Systems, Minneapolis, MN) according to the manufacturer’s protocol. The absorbance was measured using a Synergy H1 Multi-Mode Reader (Biotek). The OD of each well was used to calculate the cortisol concentration based on the values of the authentic standard curves.

### Statistical analysis

Statistical analysis was performed using a two-tailed Student’s *t-*test to compare the experimental groups. *P*-values were considered to be significant at *P* < 0.05 or *P* < 0.01.

## Supplementary information

**Additional file 1.** CRF western blot in Fig. 2e. Human DPCs were exposed to 10^− 6^ M CRF for 24 h and assessed protein expression levels of CRF using western blot.

**Additional file 2.** CRFR1 western blot in Fig. 2e. Human DPCs were exposed to 10^− 6^ M CRF for 24 h and assessed protein expression levels of CRFR1 using western blot.

**Additional file 3.** CRFR2 western blot in Fig. 2e. Human DPCs were exposed to 10^− 6^ M CRF for 24 h and assessed protein expression levels of CRFR2 using western blot.

**Additional file 4.** GAPDH western blot in Fig. 2e. Human DPCs were exposed to 10^− 6^ M CRF for 24 h and assessed protein expression levels of GAPDH using western blot.

**Additional file 5.** HGF PCR in Fig. 4a. The mRNA expression levels of HGF in cultured human DPCs with CRF treatment.

**Additional file 6.** Wnt5a PCR in Fig. 4a. The mRNA expression levels of Wnt5a in cultured human DPCs with CRF treatment.

**Additional file 7.** TGFβ2 PCR in Fig. 4a. The mRNA expression levels of TGFβ2 in cultured human DPCs with CRF treatment.

**Additional file 8.** VEGF PCR in Fig. 4a. The mRNA expression levels of VEGF in cultured human DPCs with CRF treatment.

**Additional file 9.** versican PCR in Fig. 4a. The mRNA expression levels of versican in cultured human DPCs with CRF treatment.

**Additional file 10.** ALP PCR in Fig. 4a. The mRNA expression levels of ALP in cultured human DPCs with CRF treatment.

**Additional file 11.** Actin PCR in Fig. 4a. The mRNA expression levels of Actin in cultured human DPCs with CRF treatment.

**Additional file 12.** HGF western blot in Fig. 4b. The protein expression levels of HGF in cultured human DPCs with CRF treatment.

**Additional file 13.** Wnt5a western blot in Fig. 4b. The protein expression levels of Wnt5a in cultured human DPCs with CRF treatment.

**Additional file 14.** TGFβ2 western blot in Fig. 4b. The protein expression levels of TGFβ2 in cultured human DPCs with CRF treatment.

**Additional file 15.** VEGF western blot in Fig. 4b. The protein expression levels of VEGF in cultured human DPCs with CRF treatment.

**Additional file 16.** versican western blot in Fig. 4b. The protein expression levels of versican in cultured human DPCs with CRF treatment.

**Additional file 17.** ALP western blot in Fig. 4b. The protein expression levels of ALP in cultured human DPCs with CRF treatment.

**Additional file 18.** GAPDH western blot in Fig. 4b. The protein expression levels of GAPDH in cultured human DPCs with CRF treatment.

**Additional file 19.** POMC PCR in Fig. 5b. The mRNA expression levels of POMC in cultured human DPCs with CRF treatment.

**Additional file 20.** Actin PCR in Fig. 5b. The mRNA expression levels of Actin in cultured human DPCs with CRF treatment.

**Additional file 21.** POMC and GAPDH PCR in Fig. 5c. The protein expression levels of both POMC and GAPDH in cultured human DPCs with CRF treatment.

## Data Availability

The datasets used and/or analysed during the current study available from the corresponding author on reasonable request.

## Abbreviations

CRF: Corticotropin releasing factor
ACTH: Adrenocorticotropic hormone
DPCs: Dermal papilla cells
ROS: Reactive oxygen species
POMC: Proopiomelanocortin
HFs: Hair follicles
HPA axis: Hypothalamic–pituitary–adrenal axis
CRFR: CRF receptor
GPCR: G protein coupled receptors
PKA: Protein kinase A
CREB: cAMP response element binding protein
ORSC: Outer root sheath cell
IBMX: 3-isobutyl-1-methylxanthine
