# Supplementary figures and images for: The local hypothalamic–pituitary–adrenal axis in cultured human dermal papilla cells
Source: BMC Mol Cell Biol. 2020 Jun 10;21:42. doi: 10.1186/s12860-020-00287-w (PMC7310274; doi:10.1186/s12860-020-00287-w)

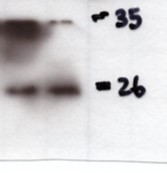

Supplement: Supplementary file 1 — Additional file 1. CRF western blot in Fig. 2e. Human DPCs were exposed to 10− 6 M CRF for 24 h and assessed protein expression levels of CRF using western blot. [file 12860_2020_287_MOESM1_ESM.jpg]

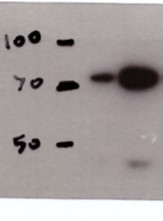

Supplement: Supplementary file 2 — Additional file 2. CRFR1 western blot in Fig. 2e. Human DPCs were exposed to 10− 6 M CRF for 24 h and assessed protein expression levels of CRFR1 using western blot. [file 12860_2020_287_MOESM2_ESM.jpg]

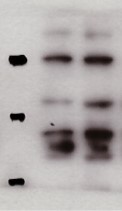

Supplement: Supplementary file 3 — Additional file 3. CRFR2 western blot in Fig. 2e. Human DPCs were exposed to 10− 6 M CRF for 24 h and assessed protein expression levels of CRFR2 using western blot. [file 12860_2020_287_MOESM3_ESM.jpg]

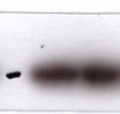

Supplement: Supplementary file 4 — Additional file 4. GAPDH western blot in Fig. 2e. Human DPCs were exposed to 10− 6 M CRF for 24 h and assessed protein expression levels of GAPDH using western blot. [file 12860_2020_287_MOESM4_ESM.jpg]

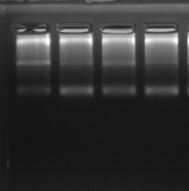

Supplement: Supplementary file 5 — Additional file 5. HGF PCR in Fig. 4a. The mRNA expression levels of HGF in cultured human DPCs with CRF treatment. [file 12860_2020_287_MOESM5_ESM.jpg]

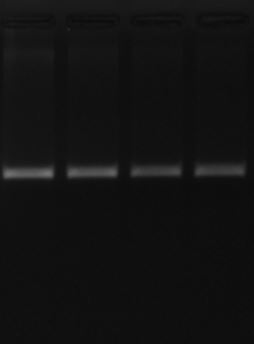

Supplement: Supplementary file 6 — Additional file 6. Wnt5a PCR in Fig. 4a. The mRNA expression levels of Wnt5a in cultured human DPCs with CRF treatment. [file 12860_2020_287_MOESM6_ESM.jpg]

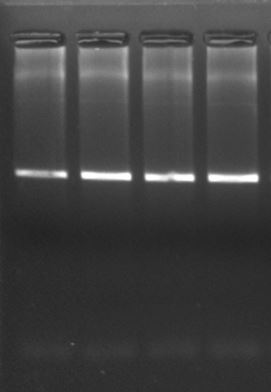

Supplement: Supplementary file 7 — Additional file 7. TGFβ2 PCR in Fig. 4a. The mRNA expression levels of TGFβ2 in cultured human DPCs with CRF treatment. [file 12860_2020_287_MOESM7_ESM.jpg]

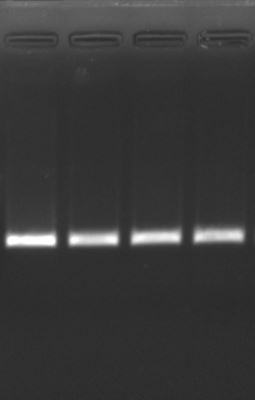

Supplement: Supplementary file 8 — Additional file 8. VEGF PCR in Fig. 4a. The mRNA expression levels of VEGF in cultured human DPCs with CRF treatment. [file 12860_2020_287_MOESM8_ESM.jpg]

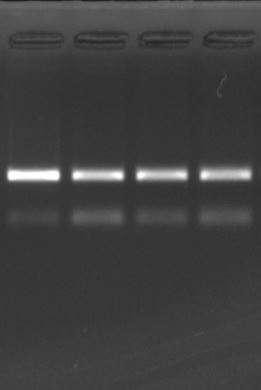

Supplement: Supplementary file 9 — Additional file 9. versican PCR in Fig. 4a. The mRNA expression levels of versican in cultured human DPCs with CRF treatment. [file 12860_2020_287_MOESM9_ESM.jpg]

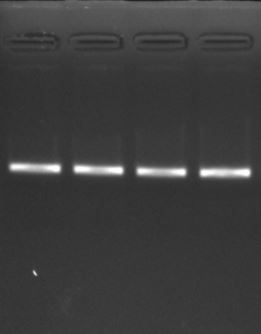

Supplement: Supplementary file 10 — Additional file 10. ALP PCR in Fig. 4a. The mRNA expression levels of ALP in cultured human DPCs with CRF treatment. [file 12860_2020_287_MOESM10_ESM.jpg]

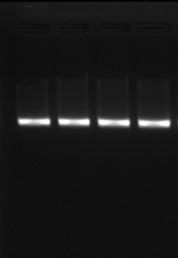

Supplement: Supplementary file 11 — Additional file 11. Actin PCR in Fig. 4a. The mRNA expression levels of Actin in cultured human DPCs with CRF treatment. [file 12860_2020_287_MOESM11_ESM.jpg]

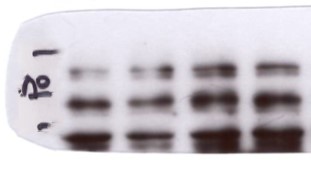

Supplement: Supplementary file 12 — Additional file 12. HGF western blot in Fig. 4b. The protein expression levels of HGF in cultured human DPCs with CRF treatment. [file 12860_2020_287_MOESM12_ESM.jpg]

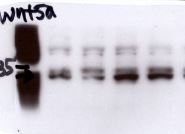

Supplement: Supplementary file 13 — Additional file 13. Wnt5a western blot in Fig. 4b. The protein expression levels of Wnt5a in cultured human DPCs with CRF treatment. [file 12860_2020_287_MOESM13_ESM.jpg]

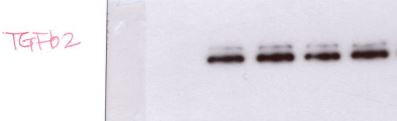

Supplement: Supplementary file 14 — Additional file 14. TGFβ2 western blot in Fig. 4b. The protein expression levels of TGFβ2 in cultured human DPCs with CRF treatment. [file 12860_2020_287_MOESM14_ESM.jpg]

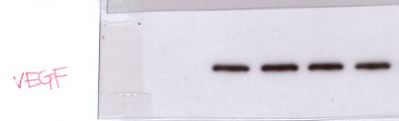

Supplement: Supplementary file 15 — Additional file 15. VEGF western blot in Fig. 4b. The protein expression levels of VEGF in cultured human DPCs with CRF treatment. [file 12860_2020_287_MOESM15_ESM.jpg]

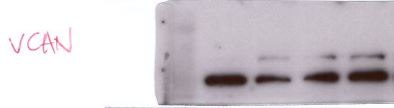

Supplement: Supplementary file 16 — Additional file 16. versican western blot in Fig. 4b. The protein expression levels of versican in cultured human DPCs with CRF treatment. [file 12860_2020_287_MOESM16_ESM.jpg]

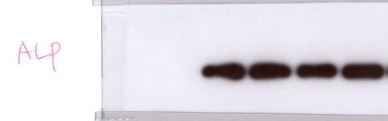

Supplement: Supplementary file 17 — Additional file 17. ALP western blot in Fig. 4b. The protein expression levels of ALP in cultured human DPCs with CRF treatment. [file 12860_2020_287_MOESM17_ESM.jpg]

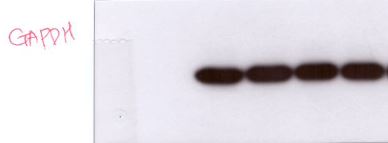

Supplement: Supplementary file 18 — Additional file 18. GAPDH western blot in Fig. 4b. The protein expression levels of GAPDH in cultured human DPCs with CRF treatment. [file 12860_2020_287_MOESM18_ESM.jpg]

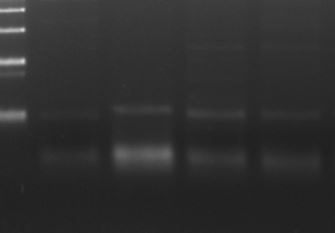

Supplement: Supplementary file 19 — Additional file 19. POMC PCR in Fig. 5b. The mRNA expression levels of POMC in cultured human DPCs with CRF treatment. [file 12860_2020_287_MOESM19_ESM.jpg]

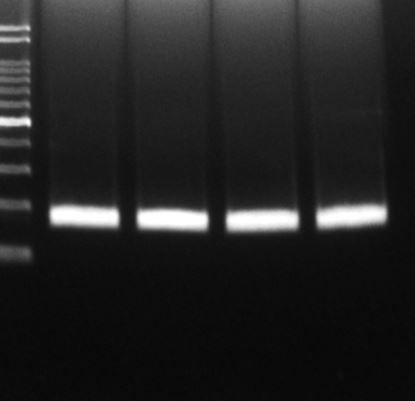

Supplement: Supplementary file 20 — Additional file 20. Actin PCR in Fig. 5b. The mRNA expression levels of Actin in cultured human DPCs with CRF treatment. [file 12860_2020_287_MOESM20_ESM.jpg]

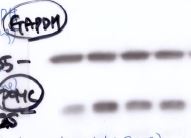

Supplement: Supplementary file 21 — Additional file 21. POMC and GAPDH PCR in Fig. 5c. The protein expression levels of both POMC and GAPDH in cultured human DPCs with CRF treatment. [file 12860_2020_287_MOESM21_ESM.jpg]
